# {6,6′-Dimeth­oxy-2,2′-[6-bromo­pyridine-2,3-diylbis(nitrilo­methyl­idyne)]­diphenol­ato}­copper(II) methanol solvate

**DOI:** 10.1107/S1600536809003316

**Published:** 2009-02-28

**Authors:** Zhen Jia

**Affiliations:** a Department of Chemistry, Dezhou University, Dezhou 253023, People’s Republic of China

## Abstract

In the title compound, [Cu(C_21_H_16_BrN_3_O_4_)]·CH_3_OH, the Cu^II^ ion is coordinated by two N [Cu—N = 1.814 (3) and 1.917 (3) Å] and two O [Cu—O = 1.805 (3) and 1.893 (3) Å] atoms from the tetra­dentate Schiff base ligand in a distorted square-planar geometry. In the crystal structure, the approximately planar Cu complex mol­ecules are paired into centrosymmetric dimers with short inter­molecular Cu⋯N distances of 3.162 (3) Å. Weak O---H...O hydrogen bonds may help to stabilize the structure.

## Related literature

For a related crystal structure, see Saha *et al.* (2007 ▶). For general background, see: Ghosh *et al.* (2006 ▶); Nayak *et al.* (2006 ▶); Singh *et al.* (2007 ▶); Yu *et al.* (2007 ▶).
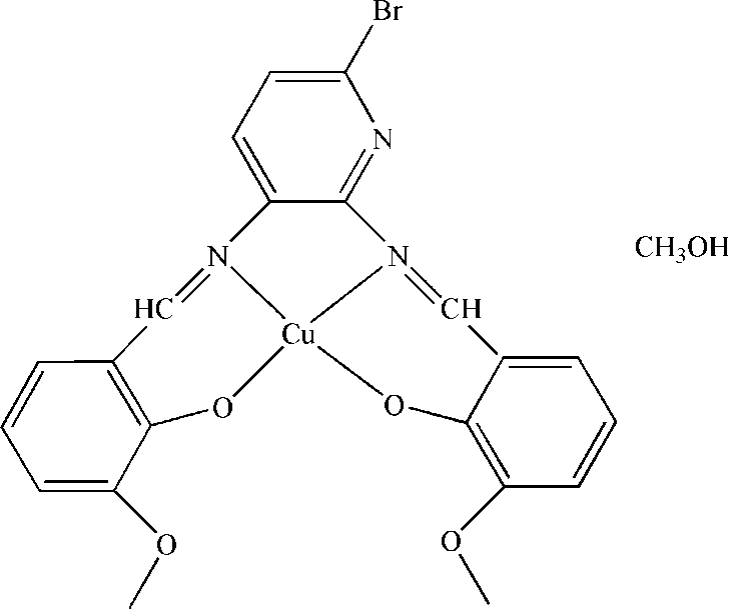


## Experimental

### 

#### Crystal data

[Cu(C_21_H_16_BrN_3_O_4_)]·CH_4_O
*M* *_r_* = 549.86
Triclinic, 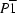

*a* = 7.4520 (8) Å
*b* = 11.5402 (13) Å
*c* = 12.9432 (14) Å
α = 104.345 (2)°
β = 96.467 (2)°
γ = 96.531 (2)°
*V* = 1059.9 (2) Å^3^
*Z* = 2
Mo *K*α radiation
μ = 2.96 mm^−1^
*T* = 293 (2) K
0.15 × 0.13 × 0.11 mm

#### Data collection

Bruker APEXII CCD area-detector diffractometer
Absorption correction: multi-scan (*SADABS*; Sheldrick, 2003 ▶) *T*_min_ = 0.665, *T*_max_ = 0.737
5332 measured reflections
3705 independent reflections
2885 reflections with *I* > 2σ(*I*)
*R*_int_ = 0.019

#### Refinement

*R*[*F* ^2^ > 2σ(*F* ^2^)] = 0.043
*wR*(*F* ^2^) = 0.139
*S* = 1.06
3705 reflections
293 parameters
H-atom parameters constrained
Δρ_max_ = 0.73 e Å^−3^
Δρ_min_ = −0.45 e Å^−3^

### 

Data collection: *APEX2* (Bruker, 2004 ▶); cell refinement: *SAINT-Plus* (Bruker, 2001 ▶); data reduction: *SAINT-Plus*; program(s) used to solve structure: *SHELXS97* (Sheldrick, 2008 ▶); program(s) used to refine structure: *SHELXL97* (Sheldrick, 2008 ▶); molecular graphics: *XP* (Sheldrick, 1998 ▶); software used to prepare material for publication: *XP*.

## Supplementary Material

Crystal structure: contains datablocks I, global. DOI: 10.1107/S1600536809003316/cv2514sup1.cif

Structure factors: contains datablocks I. DOI: 10.1107/S1600536809003316/cv2514Isup2.hkl

Additional supplementary materials:  crystallographic information; 3D view; checkCIF report

## Figures and Tables

**Table 1:** Hydrogen-bond geometry (Å, °)

*D*—H⋯*A*	*D*—H	H⋯*A*	*D*⋯*A*	*D*—H⋯*A*
O5—H5⋯O1	0.82	2.24	3.033 (5)	163
O5—H5⋯O3	0.82	2.63	3.165 (5)	124

**Fig. 1.**
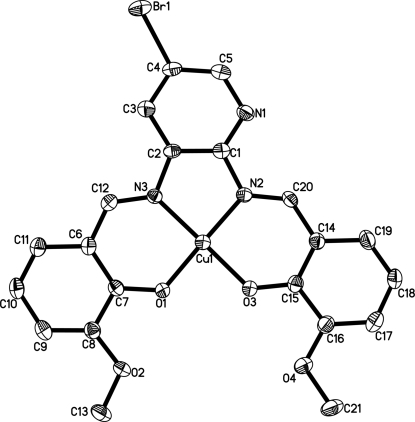
View of the title compound with the atom-labelling scheme and displacement ellipsoids drawn at the 30% probability level. All H-atoms and the methanol molecule are omitted for clarity.

## supplementary crystallographic information

### Comment

Schiff bases play an important role in the development of coordination chemistry as they readily form stable complexes with most of the transition metals, in which some could exhibit interesting properties (Yu *et al.*, 2007; Ghosh *et al.*, 2006; Singh *et al.*, 2007; Nayak *et al*., 2006). Here, we report a new Cu^II^ complex based on the tetradentate Schiff-base ligand 6-bromo-2,3-diaminopyridine-*N*,*N*'-bis (3-methoxysalicylideneimine).

The geometry and labeling scheme for the crystal structure of the title complex are shown in Figure 1. The coordination sphere for the Cu^II^ ion in the title complex is a slightly distorted square planar, in which the four positions are occupied by two N atoms and two O atoms of the Schiff-base ligand. The mean deviation from the plane formed by the two N atoms, two O atoms and the Cu ion is only 0.0329 /A%, indicative of that these five atoms are nearly coplanar. The average bond lengths of Cu—N and Cu—O are 1.866 and 1.849 /A%, respectively, which are slightly shorter than the corresponding distances in aqua-(*N*,*N*'-ethylenebis(3-methoxysalicylaldiminato)-N,*N*',*O*,*O*')copper(II) (Saha, *et al.*, 2007).

### Experimental

The Schiff base ligand was synthesized by condensation 6-bromo-2,3-diaminopyridine and 2-hydroxy-3-methoxybenzaldehyde with the ratio 1:2 in ethanol. The synthesis of the title complex was carried out by reacting Cu(ClO_4_)_2_.6H_2_O, and the Schiff-base ligand (1:1, molar ratio) in methanol. After the stirring process was continued for about 30 min at room temperature, the mixture was filtered and the filtrate was allowed to partial evaporate in air for sevral days to produce crystals suitable for X-ray diffraction with a yield about 60%.

### Refinement

All H atoms were geometrically positioned (C—H 0.93, 0.96 Å and O—H 0.82 Å), and were refined as riding, with *U*_iso_(H) = 1.2-1.5*U*_eq_(C, O).

### Crystal data

**Table:**

[Cu(C_21_H_16_BrN_3_O_4_)]·CH_4_O	*Z* = 2
*M**_r_* = 549.86	*F*(000) = 554
Triclinic, *P*1	*D*_x_ = 1.723 Mg m^−^^3^*D*_m_ = 1.723 Mg m^−^^3^*D*_m_ measured by not measured
*a* = 7.4520 (8) Å	Mo *K*α radiation, λ = 0.71073 Å
*b* = 11.5402 (13) Å	Cell parameters from 1770 reflections
*c* = 12.9432 (14) Å	θ = 3.0–24.5°
α = 104.345 (2)°	µ = 2.96 mm^−^^1^
β = 96.467 (2)°	*T* = 293 K
γ = 96.531 (2)°	Block, blue
*V* = 1059.9 (2) Å^3^	0.15 × 0.13 × 0.11 mm

### Data collection

**Table:**

Bruker APEXII CCD area-detector diffractometer	3705 independent reflections
Radiation source: fine-focus sealed tube	2885 reflections with *I* > 2σ(*I*)
graphite	*R*_int_ = 0.019
φ and ω scans	θ_max_ = 25.0°, θ_min_ = 1.8°
Absorption correction: multi-scan (*SADABS*; Sheldrick, 2003)	*h* = −8→8
*T*_min_ = 0.665, *T*_max_ = 0.737	*k* = −13→9
5332 measured reflections	*l* = −15→15

### Refinement

**Table:**

Refinement on *F*^2^	Primary atom site location: structure-invariant direct methods
Least-squares matrix: full	Secondary atom site location: difference Fourier map
*R*[*F*^2^ > 2σ(*F*^2^)] = 0.043	Hydrogen site location: inferred from neighbouring sites
*wR*(*F*^2^) = 0.139	H-atom parameters constrained
*S* = 1.06	*w* = 1/[σ^2^(*F*_o_^2^) + (0.0831*P*)^2^ + 0.2066*P*] where *P* = (*F*_o_^2^ + 2*F*_c_^2^)/3
3705 reflections	(Δ/σ)_max_ = 0.001
293 parameters	Δρ_max_ = 0.73 e Å^−^^3^
0 restraints	Δρ_min_ = −0.45 e Å^−^^3^

### Special details

**Table:**

Geometry. All e.s.d.'s (except the e.s.d. in the dihedral angle between two l.s. planes) are estimated using the full covariance matrix. The cell e.s.d.'s are taken into account individually in the estimation of e.s.d.'s in distances, angles and torsion angles; correlations between e.s.d.'s in cell parameters are only used when they are defined by crystal symmetry. An approximate (isotropic) treatment of cell e.s.d.'s is used for estimating e.s.d.'s involving l.s. planes.
Refinement. Refinement of *F*^2^ against ALL reflections. The weighted *R*-factor *wR* and goodness of fit *S* are based on *F*^2^, conventional *R*-factors *R* are based on *F*, with *F* set to zero for negative *F*^2^. The threshold expression of *F*^2^ > σ(*F*^2^) is used only for calculating *R*-factors(gt) *etc*. and is not relevant to the choice of reflections for refinement. *R*-factors based on *F*^2^ are statistically about twice as large as those based on *F*, and *R*- factors based on ALL data will be even larger.

### Fractional atomic coordinates and isotropic or equivalent isotropic displacement parameters (Å^2^)

**Table:**

	*x*	*y*	*z*	*U*_iso_*/*U*_eq_	
Cu1	0.36241 (7)	−0.01108 (4)	0.60230 (4)	0.03577 (19)	
Br1	0.23295 (10)	−0.51353 (5)	0.16747 (4)	0.0761 (3)	
O1	0.2903 (4)	0.1323 (3)	0.5995 (2)	0.0383 (7)	
O2	0.2316 (5)	0.3501 (3)	0.6365 (3)	0.0485 (8)	
O3	0.4745 (4)	0.0684 (3)	0.7448 (2)	0.0405 (7)	
O4	0.6350 (5)	0.2207 (3)	0.9307 (3)	0.0634 (10)	
O5	0.1419 (5)	0.2035 (3)	0.8114 (3)	0.0625 (10)	
H5	0.2014	0.1945	0.7612	0.075*	
N1	0.4117 (5)	−0.3542 (3)	0.4995 (3)	0.0425 (9)	
N2	0.4290 (4)	−0.1555 (3)	0.6099 (3)	0.0332 (8)	
N3	0.2583 (4)	−0.0873 (3)	0.4554 (3)	0.0314 (7)	
C1	0.3764 (5)	−0.2447 (4)	0.5091 (3)	0.0360 (9)	
C2	0.2886 (5)	−0.2069 (3)	0.4228 (3)	0.0334 (9)	
C3	0.2440 (6)	−0.2854 (4)	0.3183 (4)	0.0451 (11)	
H3	0.1896	−0.2613	0.2603	0.054*	
C4	0.2859 (6)	−0.3987 (4)	0.3081 (4)	0.0450 (11)	
C5	0.3641 (6)	−0.4313 (4)	0.3988 (4)	0.0465 (11)	
H5A	0.3850	−0.5109	0.3896	0.056*	
C6	0.1301 (5)	0.0772 (4)	0.4117 (3)	0.0359 (9)	
C7	0.1961 (5)	0.1589 (4)	0.5164 (3)	0.0342 (9)	
C8	0.1615 (6)	0.2776 (4)	0.5331 (4)	0.0397 (10)	
C9	0.0664 (6)	0.3116 (4)	0.4483 (4)	0.0478 (12)	
H9	0.0452	0.3913	0.4597	0.057*	
C10	0.0008 (6)	0.2296 (5)	0.3456 (4)	0.0501 (12)	
H10	−0.0619	0.2564	0.2919	0.060*	
C11	0.0288 (6)	0.1147 (4)	0.3262 (4)	0.0443 (11)	
H11	−0.0153	0.0602	0.2598	0.053*	
C12	0.1635 (5)	−0.0414 (4)	0.3872 (3)	0.0366 (10)	
H12	0.1166	−0.0913	0.3191	0.044*	
C13	0.2030 (8)	0.4705 (4)	0.6604 (4)	0.0583 (14)	
H13A	0.2575	0.5096	0.6117	0.087*	
H13B	0.2576	0.5108	0.7331	0.087*	
H13C	0.0743	0.4741	0.6527	0.087*	
C14	0.5858 (6)	−0.0953 (4)	0.7995 (3)	0.0379 (10)	
C15	0.5660 (6)	0.0254 (4)	0.8168 (3)	0.0372 (10)	
C16	0.6525 (6)	0.1045 (4)	0.9197 (4)	0.0446 (11)	
C17	0.7470 (7)	0.0636 (5)	1.0005 (4)	0.0547 (13)	
H17	0.7998	0.1177	1.0657	0.066*	
C18	0.7609 (7)	−0.0570 (5)	0.9823 (4)	0.0546 (13)	
H18	0.8209	−0.0859	1.0356	0.066*	
C19	0.6857 (7)	−0.1340 (5)	0.8852 (4)	0.0501 (12)	
H19	0.6988	−0.2152	0.8731	0.060*	
C20	0.5175 (6)	−0.1781 (4)	0.6964 (3)	0.0380 (10)	
H20	0.5376	−0.2576	0.6890	0.046*	
C21	0.7138 (11)	0.3026 (5)	1.0320 (5)	0.088 (2)	
H21A	0.6589	0.2793	1.0888	0.132*	
H21B	0.6927	0.3831	1.0316	0.132*	
H21C	0.8428	0.3006	1.0435	0.132*	
C22	0.2129 (10)	0.3101 (5)	0.8876 (5)	0.0784 (18)	
H22A	0.2649	0.2925	0.9523	0.118*	
H22B	0.1175	0.3582	0.9032	0.118*	
H22C	0.3060	0.3538	0.8604	0.118*	

### Atomic displacement parameters (Å^2^)

**Table:**

	*U*^11^	*U*^22^	*U*^33^	*U*^12^	*U*^13^	*U*^23^
Cu1	0.0395 (3)	0.0311 (3)	0.0352 (3)	0.0046 (2)	0.0021 (2)	0.0076 (2)
Br1	0.1168 (6)	0.0451 (3)	0.0517 (4)	0.0169 (3)	−0.0020 (3)	−0.0105 (3)
O1	0.0485 (17)	0.0308 (15)	0.0337 (15)	0.0054 (13)	−0.0022 (13)	0.0085 (12)
O2	0.066 (2)	0.0302 (16)	0.0483 (19)	0.0142 (15)	0.0021 (16)	0.0084 (14)
O3	0.0484 (17)	0.0341 (16)	0.0364 (16)	0.0062 (13)	−0.0035 (14)	0.0086 (13)
O4	0.092 (3)	0.039 (2)	0.047 (2)	0.0006 (18)	−0.0166 (19)	0.0059 (16)
O5	0.071 (2)	0.062 (2)	0.048 (2)	0.0059 (19)	0.0110 (18)	0.0016 (18)
N1	0.045 (2)	0.032 (2)	0.050 (2)	0.0079 (16)	0.0042 (18)	0.0097 (18)
N2	0.0338 (18)	0.0295 (18)	0.0352 (19)	0.0026 (14)	0.0053 (15)	0.0074 (15)
N3	0.0306 (17)	0.0291 (18)	0.0335 (18)	0.0022 (14)	0.0043 (14)	0.0078 (15)
C1	0.032 (2)	0.035 (2)	0.039 (2)	0.0020 (18)	0.0081 (18)	0.0070 (19)
C2	0.033 (2)	0.028 (2)	0.036 (2)	−0.0014 (17)	0.0079 (18)	0.0032 (18)
C3	0.050 (3)	0.038 (3)	0.045 (3)	0.007 (2)	0.002 (2)	0.007 (2)
C4	0.054 (3)	0.034 (2)	0.039 (2)	0.006 (2)	0.007 (2)	−0.003 (2)
C5	0.051 (3)	0.034 (2)	0.054 (3)	0.011 (2)	0.014 (2)	0.006 (2)
C6	0.030 (2)	0.043 (3)	0.037 (2)	0.0034 (18)	0.0054 (18)	0.017 (2)
C7	0.032 (2)	0.034 (2)	0.039 (2)	0.0039 (17)	0.0082 (18)	0.0132 (19)
C8	0.038 (2)	0.039 (2)	0.043 (3)	0.0040 (19)	0.005 (2)	0.014 (2)
C9	0.045 (3)	0.048 (3)	0.059 (3)	0.015 (2)	0.009 (2)	0.024 (2)
C10	0.047 (3)	0.059 (3)	0.050 (3)	0.015 (2)	−0.002 (2)	0.026 (3)
C11	0.040 (2)	0.051 (3)	0.041 (3)	0.005 (2)	0.001 (2)	0.015 (2)
C12	0.035 (2)	0.040 (2)	0.033 (2)	−0.0013 (18)	0.0057 (18)	0.0082 (19)
C13	0.077 (4)	0.034 (3)	0.066 (3)	0.015 (2)	0.011 (3)	0.014 (2)
C14	0.037 (2)	0.043 (3)	0.036 (2)	0.0122 (19)	0.0084 (19)	0.011 (2)
C15	0.037 (2)	0.041 (2)	0.035 (2)	0.0035 (18)	0.0057 (18)	0.0137 (19)
C16	0.046 (3)	0.045 (3)	0.040 (3)	0.002 (2)	0.000 (2)	0.010 (2)
C17	0.053 (3)	0.065 (3)	0.040 (3)	0.001 (2)	−0.005 (2)	0.011 (2)
C18	0.063 (3)	0.059 (3)	0.042 (3)	0.020 (3)	−0.007 (2)	0.017 (2)
C19	0.057 (3)	0.052 (3)	0.049 (3)	0.020 (2)	0.006 (2)	0.022 (2)
C20	0.040 (2)	0.035 (2)	0.043 (2)	0.0151 (19)	0.009 (2)	0.014 (2)
C21	0.139 (6)	0.042 (3)	0.060 (4)	−0.012 (3)	−0.032 (4)	0.002 (3)
C22	0.104 (5)	0.064 (4)	0.053 (3)	0.016 (3)	−0.008 (3)	−0.004 (3)

### Geometric parameters (Å, °)

**Table:**

Cu1—O1	1.805 (3)	C7—C8	1.392 (6)
Cu1—N2	1.814 (3)	C8—C9	1.400 (6)
Cu1—O3	1.893 (3)	C9—C10	1.423 (7)
Cu1—N3	1.917 (3)	C9—H9	0.9300
Br1—C4	1.936 (4)	C10—C11	1.332 (6)
O1—C7	1.337 (5)	C10—H10	0.9300
O2—C13	1.393 (5)	C11—H11	0.9300
O2—C8	1.398 (5)	C12—H12	0.9300
O3—C15	1.320 (5)	C13—H13A	0.9600
O4—C16	1.335 (6)	C13—H13B	0.9600
O4—C21	1.430 (6)	C13—H13C	0.9600
O5—C22	1.378 (6)	C14—C15	1.382 (6)
O5—H5	0.8200	C14—C20	1.434 (6)
N1—C1	1.298 (5)	C14—C19	1.456 (6)
N1—C5	1.366 (6)	C15—C16	1.447 (6)
N2—C20	1.331 (5)	C16—C17	1.402 (6)
N2—C1	1.430 (5)	C17—C18	1.371 (7)
N3—C12	1.317 (5)	C17—H17	0.9300
N3—C2	1.392 (5)	C18—C19	1.364 (7)
C1—C2	1.417 (6)	C18—H18	0.9300
C2—C3	1.408 (6)	C19—H19	0.9300
C3—C4	1.356 (6)	C20—H20	0.9300
C3—H3	0.9300	C21—H21A	0.9600
C4—C5	1.405 (7)	C21—H21B	0.9600
C5—H5A	0.9300	C21—H21C	0.9600
C6—C12	1.385 (6)	C22—H22A	0.9600
C6—C7	1.441 (6)	C22—H22B	0.9600
C6—C11	1.451 (6)	C22—H22C	0.9600
			
O1—Cu1—N2	177.45 (14)	C9—C10—H10	119.9
O1—Cu1—O3	85.51 (12)	C10—C11—C6	118.1 (4)
N2—Cu1—O3	93.31 (13)	C10—C11—H11	121.0
O1—Cu1—N3	93.72 (13)	C6—C11—H11	121.0
N2—Cu1—N3	87.57 (14)	N3—C12—C6	123.4 (4)
O3—Cu1—N3	177.17 (13)	N3—C12—H12	118.3
C7—O1—Cu1	127.0 (3)	C6—C12—H12	118.3
C13—O2—C8	117.2 (4)	O2—C13—H13A	109.5
C15—O3—Cu1	129.7 (3)	O2—C13—H13B	109.5
C16—O4—C21	116.2 (4)	H13A—C13—H13B	109.5
C22—O5—H5	109.5	O2—C13—H13C	109.5
C1—N1—C5	115.7 (4)	H13A—C13—H13C	109.5
C20—N2—C1	123.0 (3)	H13B—C13—H13C	109.5
C20—N2—Cu1	125.7 (3)	C15—C14—C20	119.8 (4)
C1—N2—Cu1	111.3 (3)	C15—C14—C19	118.7 (4)
C12—N3—C2	119.4 (3)	C20—C14—C19	121.3 (4)
C12—N3—Cu1	128.3 (3)	O3—C15—C14	123.0 (4)
C2—N3—Cu1	112.3 (3)	O3—C15—C16	120.7 (4)
N1—C1—C2	123.0 (4)	C14—C15—C16	116.2 (4)
N1—C1—N2	120.0 (4)	O4—C16—C17	122.9 (4)
C2—C1—N2	116.9 (4)	O4—C16—C15	113.8 (4)
N3—C2—C3	127.0 (4)	C17—C16—C15	123.3 (4)
N3—C2—C1	111.8 (3)	C18—C17—C16	119.3 (5)
C3—C2—C1	121.3 (4)	C18—C17—H17	120.3
C4—C3—C2	115.3 (4)	C16—C17—H17	120.3
C4—C3—H3	122.4	C19—C18—C17	119.1 (4)
C2—C3—H3	122.4	C19—C18—H18	120.4
C3—C4—C5	120.1 (4)	C17—C18—H18	120.4
C3—C4—Br1	118.7 (4)	C18—C19—C14	123.2 (4)
C5—C4—Br1	121.2 (3)	C18—C19—H19	118.4
N1—C5—C4	124.5 (4)	C14—C19—H19	118.4
N1—C5—H5A	117.7	N2—C20—C14	128.2 (4)
C4—C5—H5A	117.7	N2—C20—H20	115.9
C12—C6—C7	121.2 (4)	C14—C20—H20	115.9
C12—C6—C11	116.5 (4)	O4—C21—H21A	109.5
C7—C6—C11	122.3 (4)	O4—C21—H21B	109.5
O1—C7—C8	116.2 (4)	H21A—C21—H21B	109.5
O1—C7—C6	126.3 (4)	O4—C21—H21C	109.5
C8—C7—C6	117.5 (4)	H21A—C21—H21C	109.5
C7—C8—O2	113.3 (4)	H21B—C21—H21C	109.5
C7—C8—C9	118.9 (4)	O5—C22—H22A	109.5
O2—C8—C9	127.8 (4)	O5—C22—H22B	109.5
C8—C9—C10	123.0 (4)	H22A—C22—H22B	109.5
C8—C9—H9	118.5	O5—C22—H22C	109.5
C10—C9—H9	118.5	H22A—C22—H22C	109.5
C11—C10—C9	120.3 (4)	H22B—C22—H22C	109.5
C11—C10—H10	119.9		

### Hydrogen-bond geometry (Å, °)

**Table:**

*D*—H···*A*	*D*—H	H···*A*	*D*···*A*	*D*—H···*A*
O5—H5···O1	0.82	2.24	3.033 (5)	163
O5—H5···O3	0.82	2.63	3.165 (5)	124
